# Association of Neutrophil/Lymphocyte Ratio and Neutrophil/Lymphocyte Platelet Ratio With Acute Kidney Injury in Severe COVID-19

**DOI:** 10.7759/cureus.43873

**Published:** 2023-08-21

**Authors:** Edgar Bravo, Irma L Maldonado, Marco A Razo, Gloria V Martinez, Sergio Lopez

**Affiliations:** 1 Critical Care Medicine, General Hospital León, León, MEX; 2 Research Department, University of Guanajuato, León, MEX

**Keywords:** sars-cov-2 (severe acute respiratory syndrome coronavirus 2), sars-cov-2, biomarkers in acute kidney injury, acute kidney injury, covid-19, neutrophil-to-lymphocyte ratio (nlr)

## Abstract

Background

Severe disease from COVID-19 was the leading cause of admission to the emergency room and hospitalization during the pandemic in Mexico. Acute kidney injury was one of the most prevalent complications in these patients. The neutrophil/lymphocyte (NL) index and the neutrophil/lymphocyte platelet (NLP) index have previously been described as possible markers associated with complications and mortality in this disease.

Objective

To determine the association of the NL ratio and the NLP ratio in patients with acute kidney injury secondary to severe COVID-19.

Materials and methods

This is a case-control study, unpaired, of patients diagnosed with severe COVID-19 who presented or did not present acute kidney injury. On admission to the hospital, the hematological ratios were calculated, and Mann-Whitney U tests and multivariate logistic regression were performed.

Results

A total of 160 patients were included, and a difference in the NLP ratio (4.2 vs. 3.1, p = 0.001) was observed between patients with and without acute kidney injury. Additionally, the NLP ratio was the main risk variable for acute kidney injury in severe COVID-19, with an odds ratio of 2.5 and a 95% confidence interval of 1.108-5.66.

Conclusions

The NLP ratio has a moderate association and is a risk factor associated with the presence of acute kidney injury in patients with severe COVID-19.

## Introduction

Severe acute respiratory syndrome coronavirus 2 (SARS-CoV-2) is diagnosed in patients who meet the COVID-19 definition, have a positive polymerase chain reaction (PCR) test, and exhibit clinical signs of pneumonia along with one of the following: SpO2 < 94% on room air, a ratio of arterial partial pressure of oxygen to fraction of inspired oxygen (PaO2/Fio2) < 300 mmHg, a respiratory rate ≥ 30 breaths/minute, or lung infiltrates > 50% [1]. This has been the leading cause of hospital admissions during the pandemic in our country.

Acute kidney injury is a frequent condition in patients with severe COVID-19 disease [2-4]. The prevalence varies in different publications, ranging from 28% to 46% [5], and in the United States, an incidence between 37% and 40% has been reported [6,7]. Given the high prevalence of this complication, there was a need to establish a universal definition, resulting in the consensus of the Acute Disease Quality Initiative (ADQI) and the Kidney Disease Improving Global Outcomes (KDIGO) criteria for diagnosis (elevation of creatinine greater than 0.3 within 48 hours or an increase of at least 1.5 times the baseline within seven days after the initial insult, and/or urine output less than 0.5 ml/kg/hour for six hours). Acute kidney injury is categorized into three stages (I, II, and III) [7-9].

The proposed pathophysiological mechanism for renal injury in COVID-19 is related to the ability of SARS-CoV-2 to affect tissues expressing the angiotensin-converting enzyme 2 (ACE2) receptor [10-12], leading to a severe and systemic inflammatory response characterized by higher neutrophil counts, lower lymphocyte counts, coagulation cascade activation resulting in a prothrombotic state (elevated D-dimer levels and decreased platelet counts), and subsequent microcirculation dysfunction [13-17]. The kidney is an organ with high expression of toll-like receptors, making it particularly vulnerable to severe inflammatory events such as sepsis and severe COVID-19 [8]. Therefore, the study of inflammatory markers serves as an important window to obtain rapid diagnostic markers for the management of this patient population.

Among the wide range of inflammatory markers, hematologic cell indices such as the neutrophil/lymphocyte (NL) ratio and the neutrophil/lymphocyte platelet (NLP) ratio have been described as potential markers associated with or predictive of acute kidney injury in various scenarios, including cardiac surgery, sepsis, and major abdominal surgery [17,18].

Specifically, in COVID-19, it has been observed that the NL ratio increases the risk of mortality in COVID-19 patients (odds ratio: 1.02, 95% confidence interval: 1.01-1.12). Furthermore, an NLP ratio greater than 3 has been identified as a risk factor for acute kidney injury in the intensive care COVID-19 sepsis setting (odds ratio: 4.2, 95% confidence interval: 1.78-10.6) [19-21].

However, the association of these two indices with acute kidney injury in patients with severe COVID-19 disease has not been compared from the time of hospital admission. Therefore, the main objective of this study is to determine the association between the NL ratio and the NLP ratio with acute kidney injury in severe COVID-19.

## Materials and methods

Following authorization from the local ethics and research committees (HGL-CIS-2022/003, CEI-002-2022), a non-matched case-control study was conducted at Hospital General León. All patients over 18 years old with a diagnosis of severe COVID-19 [1] and a positive PCR test were included. They were admitted to the adult emergency department from January 2020 to January 2023. The two study groups were formed as follows: (a) the cases group, consisting of patients who developed acute kidney injury according to the ADQI consensus criteria [7] during the first seven days of hospital admission; and (b) the controls group, consisting of patients who did not develop acute kidney injury during the first seven days of hospital admission. Patients with chronic kidney disease (with or without renal replacement therapy), those with acute kidney injury secondary to other causes (obstructive or due to nephrotoxic drugs), and patients in shock at the time or within 24 hours of hospital admission were excluded or removed. This study adhered to the principles of good clinical practice and the Helsinki Declaration. A sample size calculation was performed, including 142 patients (71 patients in each group), with a confidence level of 95% and a power of 80%.

In all patients, hematological indices were calculated at hospital admission using absolute values from the complete blood count: (a) the NL index was calculated using the formula: neutrophils/lymphocytes; and (b) the NLP index was calculated using the formula: (neutrophils * 100)/(lymphocytes * platelets).

Additionally, the following data were collected from the medical records: severity of renal injury, use of renal replacement therapy (peritoneal dialysis, intermittent hemodialysis, or continuous slow dialysis) during hospitalization, length of hospital stay, and hospital outcome (admission to the intensive care unit, death, or discharge).

Statistical analysis

Categorical variables are described as percentages and quantitative variables are described as means (standard deviation) or medians (interquartile range) depending on the normality of the data (Kolmogorov-Smirnov test).

An independent samples t-test or Mann-Whitney U test was performed to evaluate the difference in means or medians of the hematological indices, respectively. A receiver operating characteristic (ROC) curve analysis was conducted, obtaining the area under the curve (AUC) with a 95% confidence interval using Youden's index (sensitivity + specificity - 1) to determine the optimal cutoff point for each index; furthermore, a Cohen's d test was conducted to assess the association of the NL ratio and NLP ratio with acute kidney injury.

Finally, a multiple logistic regression analysis was performed (including age, gender, type 2 diabetes mellitus, hypertension, NL index, and NLP index) to obtain odds ratios (OR) with a 95% confidence interval, identifying the main variables associated with the probability of developing acute kidney injury. In this study, a p-value ≤ 0.05 was considered statistically significant.

## Results

A total of 160 patients who met all the selection criteria were included in the final analysis. Among them, 52.5% were male, with a mean (±SD) age of 58 years (±14 years) and a BMI of 29.8 kg/m2 (±5 kg/m2). The study population consisted of 59% of patients with type 2 diabetes mellitus and 83.8% with criteria for acute respiratory distress syndrome, of which 36.2% required mechanical ventilation. Upon admission, the median (IQR) values for the NL index, NLP index, and creatinine were 8.63 (4.2-15), 4.2 (2.8-8.7), and 0.9 mg/dl (0.5-1.2 mg/dl), respectively. The prevalence of acute kidney injury was 38.7%, and the overall mortality rate was 12.5%; additionally, a mortality rate of 21% was identified (Table 1).

**Table 1:** General description of the population. Described as means (±SD) and medians (q1-q3). KDIGO: Kidney Disease Improving Global Outcomes.

	N = 160
Male, n (%)	84 (52.5)
Female, n (%)	76 (47.5)
Age, years	58 (±14)
Weight, kg	80 (±15)
Body mass index, kg/m2	29.8 (±5)
Diabetes mellitus type 2, n (%)	94 (59)
Arterial hypertension, n (%)	86 (54)
High-flow cannulas, n (%)	89 (56)
Mechanical ventilation, n (%)	58 (36.2)
Respiratory distress syndrome, n (%)	134 (83.8)
Acute kidney injury, n (%)	62 (38.7)
KDIGO I, n (%)	24 (32.4)
KDIGO II, n (%)	31 (41.9)
KDIGO III, n (%)	7 (9.4)
Renal replacement therapy, n (%)	9 (12.2)
Admission to intensive care, n (%)	27 (17)
Hospital discharge, n (%)	127 (79)
Death, n (%)	33 (21)

The study groups were as follows: 62 patients with acute kidney injury and 98 patients without acute kidney injury. There was no significant difference in gender, age, pre-existing comorbidities, prevalence of acute respiratory distress syndrome, or initiation of mechanical ventilation between the groups. However, the mortality rate in patients with acute kidney injury was 35%, while in the group without renal injury was 11% (p = <0.001, OR: 4.35, 95% CI: 1.92-9.86) (Table 2).

**Table 2:** Comparison of population study groups. Described as means (±SD) and medians (q1-q3). AKI: acute kidney injury; APACHE II: Acute Physiology and Chronic Health Evaluation II; ¥ chi-square test.

	AKI (+), n = 62	AKI (-), n = 98	Significance
Gender, n (%)			p = 0.490
Male	35 (56)	49 (50)	
Female	28 (45)	49 50)	
Age, years	60.7 (±13)	57 (±15.4)	p = 0.117
Weight, kg	80.8 (±13)	80 (±16)	p = 0.707
Body mass index, kg/m2	30 (±4.2)	30 (±5.5)	p = 0.758
Type 2 diabetes mellitus, n (%)	36 (58)	58 (60)	p = 0.888
Arterial hypertension, n (%)	34 (54)	52 (53)	p = 0.826
APACHE II scale, points	13 (9-15)	11 (7-12)	p = 0.057
High-flow cannulas, n (%)	34 (55)	55 (56)	p = 0.873
Mechanical ventilation, n (%)	28 (45.1)	30 (30.6)	p = 0.062
Respiratory distress syndrome, n (%)	51 (82.2)	83 (85)	P = 0.684
Prone position, n (%)	37 (60)	51 (52)	p = 0.344
Admission to intensive care, n (%)	13 (21)	14 (14.3)	p = 0.062
Death, n (%)	22 (35)	11 (11)	p = <0.001^¥^

**Figure 1:**
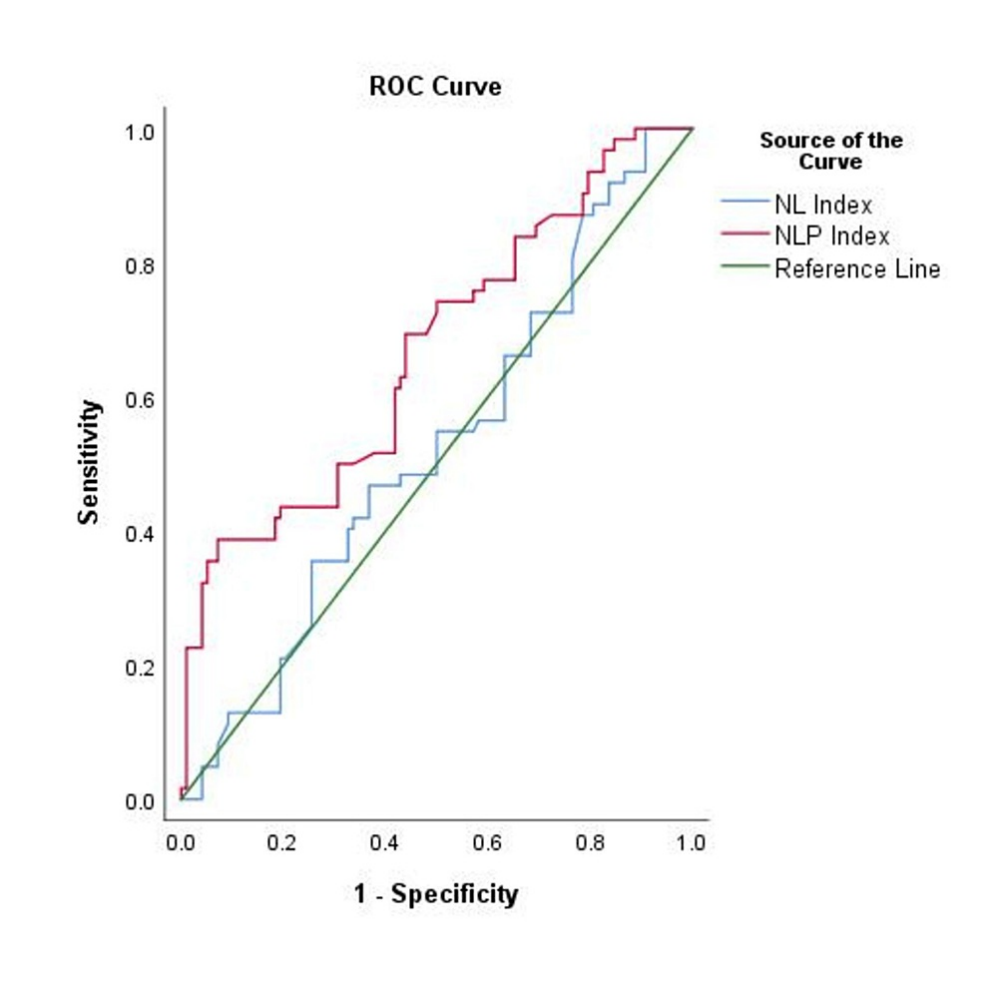
Analysis of prognostic performance. ROC curve observing the neutrophil/lymphocyte (NL) index with an AUC of 0.523 and the neutrophil/lymphocyte-platelet (NLP) index with an AUC of 0.670. The latter had a global quality analysis of 0.58 and the optimal cutoff point was determined to be 5 (Youden index of 3.56). ROC: receiver operating characteristic; AUC: area under the curve.

Regarding the primary objective, there was no significant difference in the NL index between patients with and without acute kidney injury (8.6 vs. 8.5, p = 0.619). However, the NLP index showed a significant difference on the first day between the groups (4 vs. 3.1, p = 0.045). The ROC curve analysis revealed an AUC of 0.523 (95% CI: 0.433-0.614, p = 0.621) for the NL index and 0.670 (95% CI: 0.532-0.708, p = <0.001) for the NLP index (Figure 1). The optimal cutoff points for the NL index and NLP index were 10 (Youden's index of 1.13) and 5 (Youden's index of 3.56), respectively; in addition, the NL ratio showed a mild association with acute kidney injury (Cohen's d of 0.31), and the NLP ratio exhibited a moderate association with acute kidney injury (Cohen's d of 0.61).

In the multivariable regression analysis, the NLP index was identified as a risk factor for the development of acute kidney injury in patients with severe COVID-19 (OR: 2.5, 95% CI: 1.108-5.66).

**Figure 2:**
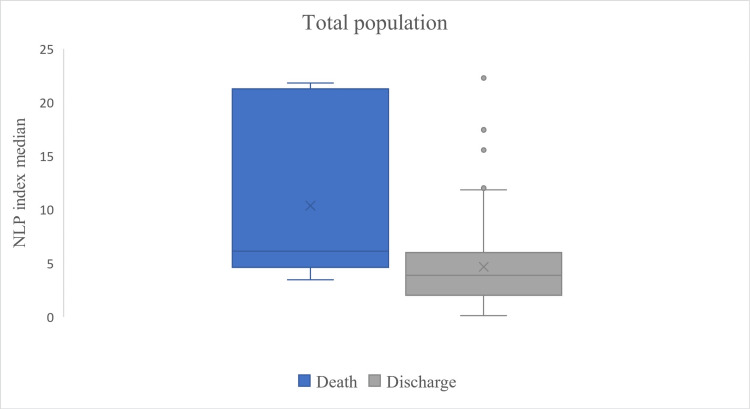
Comparison of the total population at the outcome. Box and whisker plot showing a median neutrophil/lymphocyte-platelet (NLP) index of 6.1 (4.6-21) in those who were deceased and 3.8 (2-6) in those who were discharged. The Mann-Whitney U test yielded a p-value of 0.003.

**Figure 3:**
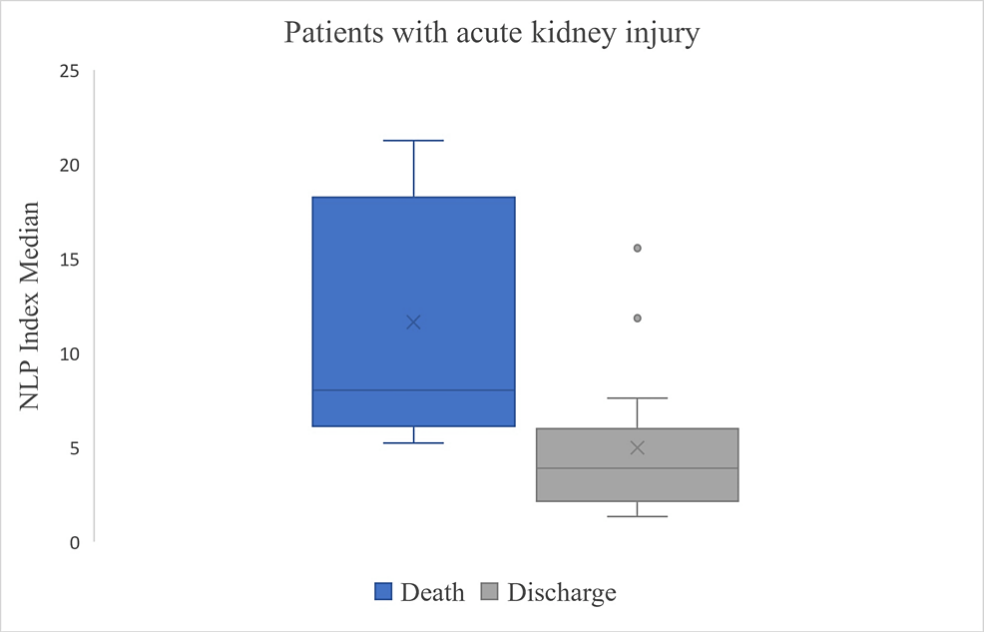
Comparison of the population with acute kidney injury at the outcome. Box and whisker plot displaying a median neutrophil/lymphocyte-platelet (NLP) index of 8 (6.1-17.5) in patients with acute kidney injury who were deceased and 3.9 (2.1-5.5) in those who were discharged. The Mann-Whitney U test yielded a p-value of <0.001.

Finally, when analyzing the outcomes of the study population, a significant difference was observed in the medians of the NLP index between patients discharged from the hospital and those who died (6.1 vs. 3.1, p = 0.002, Figure 2). Similarly, among patients with acute kidney injury, the NLP index was higher in those who died compared to those who survived (8 vs. 3.9, p = 0.001, Figure 3). Additionally, an NLP index < 5 was associated with a decreased risk of mortality in patients with acute kidney injury (OR: 0.417, 95% CI: 0.260-0.669).

## Discussion

In this study, we found that the NLP index is an independent risk factor for acute kidney injury in patients with severe COVID-19, whereas the NL index did not show a significant association. Furthermore, an increase in the NLP index was associated with a higher probability of mortality during hospitalization, regardless of the presence of acute kidney injury.

These findings are important as acute kidney injury involves a complex interplay of inflammatory, immunological, microcirculatory, and hemodynamic components that contribute to organ dysfunction [22,23]. In recent years, various predictors and predisposing factors for the development of acute kidney injury have been studied, including the NL ratio and the NLP ratio [21-24].

Our results are consistent with the study by Cheng et al. [10], which reported higher leukocyte counts (7.2 vs. 9.5, p = 0.005), lower lymphocyte counts (0.9 vs. 0.7, p = 0.015), and decreased platelet counts (216 vs. 191, p = 0.014) in patients with COVID-19 who developed acute kidney injury. Similar findings were also reported by Contreras-Chavez et al. [20] in a cohort of critically ill patients with COVID-19, where 68.4% of those with acute kidney injury had an NLP index greater than 3, which was a significant risk factor in both the univariate (OR: 5.59, 95% CI: 2.4-12.7) and multivariate analyses (OR: 4.25, 95% CI: 1.7-10.1).

It is worth noting that in our study, the NL index was not associated with acute kidney injury in these patients. These findings differ from those reported by Chen et al. [25], where an NL index in the highest quartile (greater than 20.5) was associated with acute kidney injury (OR: 1.57, 95% CI: 1.27-1.80) and increased mortality risk at 180 days (HR: 1.14, 95% CI: 1.02-1.28). Similarly, Gameiro et al. [26] found higher NL indices in patients with COVID-19 and acute kidney injury (4.9 vs. 7.8, p = 0.001, OR: 1.13, 95% CI: 1.05-1.28) and in those who died (8.5 vs. 6.05, p = 0.023, OR: 1.06, 95% CI: 1.01-1.13).

Finally, in other important findings from our study, we observed that the NLP index was associated with mortality in the overall study population and patients with acute kidney injury. This is consistent with the findings of Gameiro et al. [21], where a higher NLP index at admission to the intensive care unit was associated with mortality in patients with sepsis-related acute kidney injury (AUC: 0.565, 95% CI: 0.515-0.615, p = 0.034).

The main limitations of the study were: (1) being a single-center and retrospective study; (2) due to the nature of the population and the study's timeline, a matched case-control study could not be conducted to control factors like age, gender, or BMI; (3) despite being limited to patients without shock status, being a retrospective study meant no control over other confounding variables such as the occurrence of infectious processes in the first seven days of evaluation, patients with ARDS who exhibited kidney-lung interaction, or hematological alterations caused by the use of steroids or IL-6 blockers, which could have introduced bias in the causal relationship between the measured hematological associations and acute kidney injury; and (4) similarly, being a retrospective study, it was not possible to ensure proper standard care in the follow-up of mechanically ventilated patients outside the intensive care setting.

## Conclusions

The NLP ratio has a moderate association with acute kidney injury and is a potential risk factor in patients with severe COVID-19. Furthermore, an increase in the NLP ratio raises the risk of in-hospital mortality in patients with severe COVID-19, whether or not they have acute kidney injury.
